# Self-Stabilizing Covalent Ligand Targets Bacterial Phosphatidylethanolamine and Enhances Antibiotic Efficacy

**DOI:** 10.3390/pharmaceutics18010071

**Published:** 2026-01-05

**Authors:** Keita Masuda, Yasuhiro Nakagawa, Quentin Boussau, Emilie Chabert, Tsukuru Masuda, Jerome Bonnet, Tatsuya Inukai, Shigeki Nakamura, Madoka Takai, Diego Cattoni, Horacio Cabral

**Affiliations:** 1 Department of Bioengineering, Graduate School of Engineering, The University of Tokyo, 7-3-1 Hongo, Bunkyo-ku, Tokyo 113-8656, Japan; 2 Centre de Biologie Structurale, CNRS UMR 5048, INSERM U1054, Université de Montpellier, 29 Rue de Navacelles, 34090 Montpellier, France; 3 Department of Microbiology, Tokyo Medical University, 6-1-1 Shinjuku, Shinjuku-ku, Tokyo 160-8402, Japan; 4 Research Center for Water Environment Technology, School of Engineering, The University of Tokyo, 7-3-1 Hongo, Bunkyo-ku, Tokyo 113-8656, Japan

**Keywords:** targeted therapy, phosphatidylethanolamine, Schiff base, Gram-negative bacteria, *Escherichia coli*, Gemifloxacin

## Abstract

**Background/Objectives**: Discriminating bacterial from mammalian membranes remains a central challenge in antibiotic design. Bacterial membranes are enriched in phosphatidylethanolamine (PE), a lipid normally absent from the outer leaflet of mammalian cells, providing a signature for selective molecular engagement. We report a compact covalent ligand, 6-dimethylamino-4-ketohexanoic acid (DMAX), which targets PE via Schiff base formation, leveraging its tertiary amine to facilitate the reaction and strengthen ionic binding with the phosphate group. **Methods**: The reactivity of DMAX and PE was evaluated by computational simulations, and their interaction was examined by spectroscopic analyses (NMR and FT-IR) and an artificial membrane assay. The targeting ability of DMAX for live bacteria was determined by microscopy study, and its applicability to therapeutic system was tested in vitro under washed conditions that mimic rapid in vivo clearance. **Results**: Spectrometric analyses revealed the selective covalent interaction of DMAX and PE, consistent with the simulated results. Fluorescently labeled DMAX selectively binds PE-enriched model membranes and efficiently recognizes Gram-negative bacteria while sparing mammalian cells. Conjugation of DMAX to Gemifloxacin (Gem) significantly enhanced antibiotic efficacy by 10-fold compared with free Gem, even after rapid drug clearance, while maintaining safety in mammalian cells. **Conclusions**: These results identify DMAX as an efficient and versatile PE-targeting platform, enabling selective membrane anchoring to advance precision antibiotic strategies.

## 1. Introduction

Enhancing bacterial-membrane targeting has potential for improving the potency and selectivity of antibiotics [1]. However, current targeting approaches, such as antibody-drug conjugates [2,3] and antimicrobial peptides [4], face significant translational barriers, including high production costs [5,6], limited stability [7,8,9,10], poor pharmacokinetics [11,12], and restricted tissue penetration [13]. These challenges highlight the need for alternative mechanisms that enable efficient and selective recognition of bacterial membranes.

**Figure 1:**
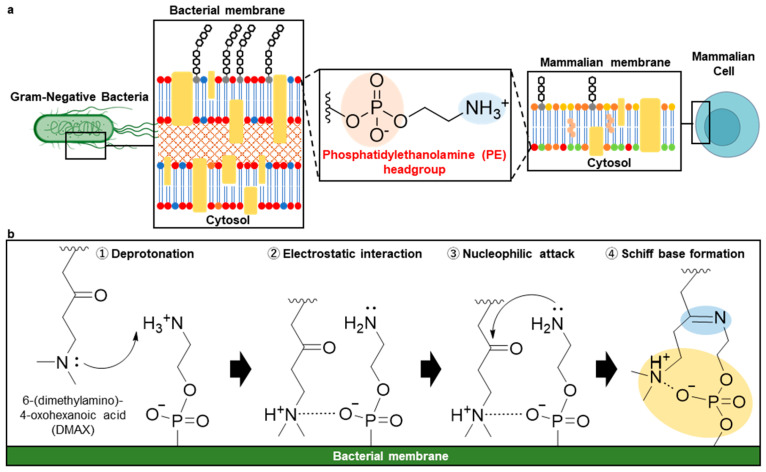
(**a**) Distribution of PE in mammalian and bacterial membranes. In mammalian cells, PE is predominantly located on the inner leaflet of the plasma membrane. In contrast, Gram-negative bacteria display PE throughout their membranes including the outer membrane. (**b**) DMAX is designed to form a reversible Schiff base with the primary amine of the phosphatidylethanolamine headgroup, with its tertiary amine transiently deprotonating the PE amine to promote imine formation. Subsequent protonation enables electrostatic association with the PE phosphate, pre-organizing and stabilizing the complex.

A key feature distinguishing bacterial membranes from their eukaryotic counterparts is their lipid composition [14]. In particular, in Gram-negative bacteria such as *Escherichia coli* (*E. coli*), phosphatidylethanolamine (PE) comprises approximately 70–80% of the total phospholipids [15,16] and is enriched in its outer membrane [17] (Figure 1a). In contrast, mammalian plasma membranes exhibit transbilayer asymmetry, with PE largely restricted to the inner cytosolic leaflet, while phosphatidylcholine (PC) and sphingomyelin (SM) are enriched in the outer leaflet [18]. This compositional and topological contrast of bacteria and mammalian cells creates a chemical signature that can be harnessed for bacterial targeting. Unfortunately, current PE-binding scaffolds are limited by suboptimal selectivity, potential toxicity, and relatively large molecular size and complexity. Despite this opportunity, existing PE-binding scaffolds suffer from important drawbacks. Peptide-based PE binders such as duramycin and cinnamycin exhibit strong affinity for PE [19], but act through membrane-disruptive mechanisms, which can lead to cytotoxicity toward mammalian cells [20]. Crown ethers have also been explored as PE-targeting motifs. However, analogs with higher PE affinity frequently show increased toxicity toward mammalian membranes, indicating a trade-off between binding strength and selectivity [21,22,23]. Cyclotides represent another class of PE-recognizing agents, but their activity is commonly associated with hemolytic membrane disruption, further limiting their therapeutic applicability [24,25]. Together, to tackle these limitations, compact, non-lytic, and chemically well-defined PE-targeting ligand with improved selectivity and safety profiles is required.

To address these limitations, we developed a compact and reversible covalent ligand, 6-dimethylamino-4-ketohexanoic acid (DMAX), designed to selectively engage PE through coordinated covalent and electrostatic interactions. The driving hypothesis is that DMAX forms a reversible Schiff base with the primary amine of PE. The proximal tertiary amine of DMAX serves as an intramolecular base, transiently deprotonating the PE amine to increase its nucleophilicity and promote imine formation. Once protonated, the tertiary amine electrostatically associates with the phosphate in the PE headgroup, stabilizing the complex (Figure 1b). This coordinated base and electrostatic anchoring mechanism is expected to allow efficient PE ligation, driving selective bacterial membrane engagement and antibiotic potentiation. Moreover, because Schiff base formation is reversible in physiological environments [26,27,28,29], DMAX-drug conjugates can dynamically associate with and dissociate from PE-rich bacterial membranes. This reversible membrane anchoring increases local drug residency at the bacterial surface while permitting antibiotic release, thereby enhancing the likelihood of bacterial uptake. Importantly, because PE is not exposed on the outer leaflet of healthy mammalian cell membranes, DMAX is expected to preferentially target bacterial cells while minimizing off-target interactions [14,18]. We validated this design through in silico reactivity mapping, spectroscopic characterization of PE binding, and fluorescence-based studies in model membranes, bacteria, and mammalian cells. Finally, conjugation of DMAX to Gemifloxacin (Gem) enhanced antibacterial efficacy in vitro, establishing DMAX as a PE-selective ligand for membrane-targeted antibiotic potentiation.

## 2. Materials and Methods

### 2.1. Materials

Dipotassium deuterium phosphate (98 atom% D, K_2_DPO_4_), 6-dimethylamino-4-ketohexanoic acid hydrochloride (DMAX), paraformaldehyde (PFA), fluorescein sodium salt (fluorescein), penicillin–streptomycin, Dulbecco’s modified Eagle’s medium (high glucose, DMEM), and RPMI-1640 medium (RPMI) were purchased from Sigma-Aldrich Co. LLC (St. Louis, MO, USA). O-phosphoryl ethanolamine (OPE), fluorescein 5-isothiocyanate (isomer I, FITC), ethylenediamine anhydrous (EDA), n-octylamine hydrochloride (OA), tetradecylamine (TA), dodecyl dihydrogen phosphate (DP), 1,2-dioleoyl-sn-glycero-3-phosphoethanolamine (PE), 1,2-dioleoyl-sn-glycero-3-phosphocholine (PC), oxalyl chloride, 4-(4,6-dimethoxy-1,3,5-triazin-2-yl)-4-methylmorpholinium chloride (DMT-MM), and deuterium oxide (99.8 atom% D, D_2_O) were purchased from Tokyo Chemical Industry Co., Ltd. (Tokyo, Japan). Potassium bromide (KBr), triethylamine (TEA), benzene, dichloromethane (super dehydrated, DCM), N,N-dimethylformamide (super dehydrated, DMF), dimethyl sulfoxide (DMSO), diethyl ether, methanol, phosphate-buffered saline without calcium and magnesium [D-PBS(–), PBS], sodium chloride (NaCl), fetal bovine serum (FBS, Spain origin), chloroform, glycerol, and agar powder were purchased from FUJIFILM Wako Pure Chemical Corporation (Osaka, Japan). Gem mesylate was purchased from LKT Laboratories, Inc. (St. Paul, MN, USA). Phosphatidylserine (PS) was purchased from Funakoshi Co., Ltd. (Tokyo, Japan). Hoechst 33342 solution (Hoechst) and Cell Counting Kit-8 (CCK-8) were purchased from Dojindo Laboratories (Kumamoto, Japan). Tryptone and yeast extract were purchased from Nacalai Tesque, Inc. (Kyoto, Japan). Sodium 4-oxopentanoic acid (OXA) was purchased from Kanto Chemical Co., Inc. (Tokyo, Japan). 5-(Dimethylamino)-5-oxopentanoic acid (DMAO) was purchased from Key Organics Ltd. (Camelford, UK). PD-10 desalting columns were purchased from Cytiva KK (Tokyo, Japan). DMSO-d_6_ was purchased from Cambridge Isotope Laboratories, Inc. (Tewksbury, MA, USA). All hazardous reagents were handled in a fume hood to ensure safety.

### 2.2. Instruments

Fourier-transform infrared spectroscopy (FT-IR) was performed using an FT/IR-6300 (JASCO Corporation, Tokyo, Japan). Lyophilization was conducted with an FDU-2110 freeze dryer (Tokyo Rikakikai Co., Ltd., Tokyo, Japan). Confocal laser scanning microscopy (CLSM) images were obtained using LSM780 and LSM880 systems (Carl Zeiss, Oberkochen, Germany) with excitation lasers at 405, 518, and 633 nm. Bacterial concentrations were measured by optical density (OD_600_) using a cell density meter (CO8000 Biowave, Biochrom, Holliston, MA, USA) or a microplate reader (Cytation 3, Agilent Technologies Japan, Ltd., Tokyo, Japan). Solvents were evaporated using a rotary evaporator (N-1300, Tokyo Rikakikai Co., Ltd.) equipped with a low-temperature circulation bath (CCA-1112A), solvent recovery unit (DPE-1150), constant-temperature oil bath (OBS-2200), and diaphragm vacuum pump (NVP-2100). NMR spectra were obtained with a JMTC-400/54/SS spectrometer (JEOL, Tokyo, Japan). Mass spectra were obtained with an ESI-time-of-flight mass spectrometry (TOF-MS, Bruker, MA, USA).

### 2.3. Cells

*E. coli* NBRC 3972 (Biological Resource Center, NITE, Chiba, Japan) and mKate2 expressing *E. coli* (mKate^+^ *E. coli*) EcNDfim_mKATE2 Dmat_lux (constructed in the Department of Microbial Biotechnology, CNB-CSIC, Madrid, Spain) [30] were preserved in sterile glycerol stock (50% glycerol in deionized water) and cultured in sterilized LB medium containing NaCl (1%), tryptone (1%), and yeast extract (0.5%). HEK293, RAW264.7, and B16F10 cells were purchased from RIKEN BioResource Center (Tsukuba, Japan) and maintained in DMEM (pH 7.4) supplemented with 10% FBS and 1% penicillin-streptomycin. DC2.4 cells were purchased from RIKEN BioResource Center (Tsukuba, Japan) and cultured in RPMI-1640 medium (pH 7.4) supplemented with 10% FBS and 1% penicillin-streptomycin. All mammalian cells were maintained at 37 °C in a humidified atmosphere containing 5% CO_2_. In addition to OD_600_, the number of the viable bacteria was determined by colony forming units (CFU).

### 2.4. Computational Method

Computational analyses were performed using the NorthWest Computational Chemistry Package (NWChem, version 7.2.2, Pacific Northwest National Laboratory, Richland, WA, USA) for quantum-chemical calculations, Open Babel (version 3.1.1) for molecular file conversions, Avogadro (version 1.2.0) for molecular model preparation, and VESTA (version 3, National Institute for Materials Science, Tsukuba, Japan) for visualization of volumetric electron density maps. Python (version 3.12.11, Python Software Foundation, Wilmington, DE, USA) served as the primary environment for data handling and analysis, with NumPy (version 2.3.2) providing numerical support. These tools were installed and executed on a Linux-based workstation configured with an MPI build of NWChem.

Molecular structures were first drawn manually and saved in .mol format. Three-dimensional geometries were optimized using the NorthWest Computational Chemistry Package (NWChem, version 7.2.2) [31]. We defined the following molecular models: i. ethyl-DMAX (EDMAX) and EDMAXH model the experimental DMAX conjugated with ethylamine and its protonated analog with minimal scaffolds capturing the ketone and tertiary-amine behavior. ii. [(2R)-3-[2-aminoethoxy(hydroxy)phosphoryl]oxy-2-propanoyloxypropyl] propanoate (AEPP) models a simplified PE-like phosphate ester bearing a primary amine with short lipid tails. AEPPH models the protonated form AEPP. iii. 1,2-dibutyrylphosphatidylcholine (DPC) models a phosphatidylcholine with short lipid tails as a negative biological control for amine-based nucleophilic chemistry. Site-selective reactivity for EDMAX, EDMAXH, AEPP, AEPPH, and DPC was calculated by NWChem [32]. Single-point electronic structures for the neutral (N), N + 1, and N − 1 states were calculated by NWChem at B3LYP/6-31G*. Electron densities and Fukui indices (f^+^, f^−^, Δf) were calculated by NWChem and post-processed by Python scripts; condensed (per-atom) indices were calculated by Mulliken population analysis with Löwdin checks [33]. Volumetric f^+^/f^−^ [34] maps were prepared by DPLOT and visualized by VESTA and Avogadro. Molecular formats and 3D coordinates were prepared by Open Babel.

### 2.5. Spectrometry Analysis

To test Schiff base formation and the role of electrostatics, DMAX, OXA, or DMAO (each 0.28 M) was mixed with OPE or OA (0.28 M) in deionized water and incubated at 37 °C for 1 h. Samples were freeze-dried and analyzed by FT-IR: 1 mg sample was mixed with 100 mg KBr, pressed into pellets, and analyzed on an FT/IR-6300. Schiff bases were evaluated by the emergence of a band at 1660~1680 cm^−1^ (C=N stretch) [35]. For ^13^C-NMR and ^1^H-NMR, DMAX, OXA, or DMAO (0.28 M) was dissolved in D_2_O, mixed with OPE or OA (0.28 M), incubated 1 h at 37 °C, and measured at 37 °C; disappearance of the DMAX ketone carbon at ~225 ppm indicated imine formation [36]. Salt dependence was assessed by adding NaCl (500 mM) to DMAX-OPE mixtures prior to incubation.

### 2.6. Preparation of FITC-Conjugates

FITC was conjugated with DMAX through an EDA linker, following reported procedures with modifications [37,38]. FITC (100 mg, 0.25 mmol) was dissolved in DMF (1 mL) and added dropwise to EDA (5 mL). The reaction was stirred for 24 h at room temperature. Solvent was removed under reduced pressure, and crude FITC-EDA was precipitated in diethyl ether, collected by filtration, and dried under vacuum.

FITC-EDA (0.05 mmol, 22 mg) was then conjugated to DMAX (52 mg, 0.25 mmol) in methanol (3 mL) and water (1 mL) by adding DMT-MM (139 mg, 0.5 mmol) to the mixture and stirring for 24 h at room temperature. The product was purified using a PD-10 column (water as eluent) and analyzed by ^1^H-NMR to confirm conjugation. DMAX-FITC or fluorescein was first dissolved in DMSO, diluted 100-fold in PBS, 0.22-µm filtered, and adjusted to RFU = 4500 before use.

### 2.7. Binding to Lipid Coated Surfaces

Glass substrate can be functionalized with lipid bilayer to assess the surface properties [39,40]. Glass-bottom 96-well plates were coated with 20 µL of lipid solutions (5 mg mL^−1^ in chloroform). Lipid compositions included PC:PE at ratios of 1:0, 1:4, 2:3, 1:1, 4:1, and 0:1. Control coatings included 20 µL of lipid solutions PS (5 mg mL^−1^), 6 µL of lipid solutions TA (5 mg mL^−1^) or DP (5 mg mL^−1^). Plates were vacuum-dried for 3 h to remove solvent. DMAX-FITC or free fluorescein solution was added to each well, incubated at 37 °C for 1 h with gentle shaking (80 rpm), and washed six times with PBS. Fluorescence was measured on a plate reader. FITC excitation/emission were 495/540 nm. The data were normalized to the non-coated wells to get the relative fluorescence intensity, and the differences in the relative fluorescence of DMAX-FITC and free fluorescein are presented.

### 2.8. Binding to Bacteria

*E. coli* (OD_600_ = 0.5, $3.2\;\times \;10^{8}$ CFU/mL) were centrifuged, resuspended in PBS containing DMAX-FITC or fluorescein, and incubated at 37 °C for 1 h. Cells were washed twice (5000 rpm, 5 min), counterstained with Hoechst, and imaged by CLSM. Colocalization was quantified from mean FITC intensity in 40 bacterial regions of interest (ROIs), and normalized to fluorescein controls by ImageJ software (version 1.54g, National Institutes of Health, Bethesda, MD, USA). For comparison with mammalian cells, the result was analyzed with 10 ROIs.

### 2.9. Binding to Mammalian Cells

HEK293, RAW264.7, DC2.4, and B16F10 cells (5 × 10^3^/well) were seeded in 48-well chamber slides and incubated for 24 h. Cells were fixed with 4% PFA (60 min), washed, quenched with FBS-containing medium, and stained with Hoechst. DMAX-FITC or fluorescein in PBS was added for 1 h at 37 °C. After two washes, CLSM imaging was performed. FITC intensity was quantified from 10 ROIs per cell, normalized to fluorescein controls.

### 2.10. Preparation of DMAX-Gem Conjugate

DMAX (50 mg, 0.24 mmol) and Gem (58 mg, 0.15 mmol) were co-freeze-dried with benzene to remove residual water. DMAX (50 mg, 0.24 mmol) was suspended in DCM (2.5 mL), activated with oxalyl chloride (1 mL, 12 mmol) for 6 h [41], and dried under vacuum. The intermediate (DMAX-Cl) was dissolved in dry DCM (5 mL) and reacted with Gem (58 mg, 0.15 mmol) in DCM (5 mL) with TEA (0.042 mL, 0.30 mmol) for 24 h at room temperature. Products were washed with DCM, dried, and confirmed by FT-IR and ^1^H-NMR.

### 2.11. Bacterial Growth Inhibition Assay

mKate^+^ *E. coli* bearing cytoplasmic mKate was grown overnight, and 1:500 dilution was performed and bacteria were grown to reach OD_600_ = 0.2 ($9.0\;\times \;10^{7}$ CFU/mL). Then the bacteria were treated with Gem (positive control) or DMAX-Gem at 0.86, 8.6, 17.2, and 86 μM, or PBS. After 3 min exposure, the samples were split into two. Half of the samples were washed (centrifugation, 5000 rpm, 3 min, ×3), while the others remained unwashed. Growth was assessed by absorbance at 600 nm and CFU assays. Serial 10-fold dilutions were spotted on agar plates (3 replicates/dilution) and incubated at 37 °C for 15 h. Colonies were counted from the lowest dilution with discrete colonies.

### 2.12. Cytotoxicity of the DMAX-Gem Against Mammalian Cells

HEK293, RAW264.7, DC2.4, and B16F10 cells (2 × 10^3^/well, 96-well plates) were incubated for 24 h, then treated with serial dilutions of drugs (86, 17.2, 1.72, 0.86, 0.172, 0.086, and 0.0172 μM) or PBS and cultured for 24 h. The treatment includes DMAX-Gem, DMAX modified Gem, or free Gem, as a positive control with some native toxicity [42]. After 3 h incubation with CCK-8 reagent (10 µL), absorbance at 450 nm was measured. The data were normalized to the control group (PBS) to obtain the cell viabilities.

### 2.13. Statistical Analysis

For two-group comparisons, one-tailed Student’s *t*-tests were used. For analysis of more than 2 groups, one-way ANOVA was used to determine an omnibus difference followed by directional pairwise one-tailed *t*-tests with Holm–Šidák correction for multiple comparisons. The data were reported as mean ± SEM.

## 3. Results and Discussion

### 3.1. Computational Analysis

**Figure 2:**
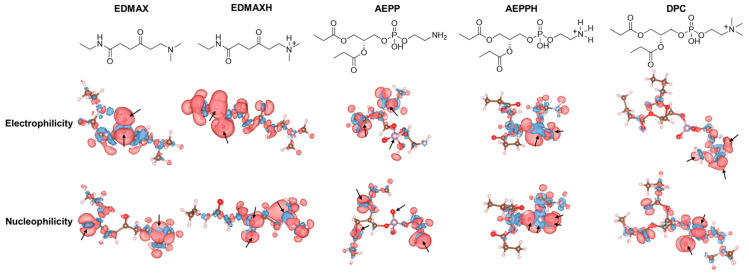
Chemical structures of derivatives of targeting ligands and lipids with electrophilic and nucleophilic lobes derived from Fukui function analysis; red lobes indicate reactive regions and blue lobes unreactive ones.

To rationalize the selection of DMAX as a PE-targeting ligand, we performed an in silico reactivity mapping based on condensed Fukui functions. This analysis allowed us to quantify the local electrophilic and nucleophilic tendencies of DMAX and PE under their relevant protonation states, clarifying which atoms are chemically predisposed to participate in reversible covalent bonding. The results showed that the ketone carbonyl carbon is the most electrophilic site in the DMAX conjugated model, i.e., ethylamine-DMAX (EDMAX) (condensed f^+^ ≈ 0.10–0.15), while the dimethylamine nitrogen is nucleophilic when neutral (f^−^ ≈ 0.03–0.06) (Figure 2). Protonation of the dimethylamine attenuates the amine nucleophilicity (f^−^ ≲ 10^−3^) yet preserves the ketone electrophilicity (carbonyl f^+^ ≈ 0.10–0.15) in the protonated EDMAX model (EDMAXH). In a model of the phosphatidylethanolamine headgroup, i.e., [(2R)-3-[2-aminoethoxy(hydroxy)phosphoryl]oxy-2-propanoyloxypropyl] propanoate (AEPP), the nucleophilicity concentrates at the primary amine (f^−^ ≈ 0.03–0.06), and it is lost upon protonation (f^−^ ≲ 10^−3^). When protonated (AEPPH), the amine is essentially inactive (f^+^ = 0.0038, f^−^ = 0.0010), while the phosphate oxygens remain nucleophilic (oxo-O f^−^ = 0.138; protonated P-O f^−^= 0.118; tail-side P-O f^−^ = 0.106) and the phosphorus is electrophilic (P f^+^ = 0.212). As a control, we modeled the phosphocholine headgroup of PC using 1,2-dibutyrylphosphatidylcholine (DPC), and confirmed that it lacks a usable amine nucleophile (quaternary ammonium f^−^ ≲ 10^−3^). These calculations show that the DMAX carbonyl retains its role as the dominant electrophilic site, while the PE amine acts as the primary nucleophile in its neutral form but diminishes reactivity upon protonation. This behavior is consistent with Schiff base formation and highlights the role of phosphate groups in stabilizing the complex.

### 3.2. Interaction of DMAX and PE

**Figure 3:**
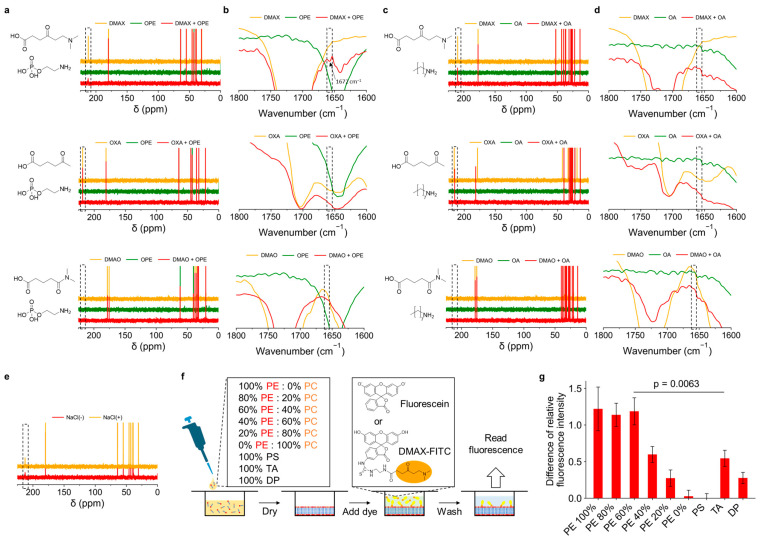
(**a**) ^13^C-NMR spectra of single compounds and mixtures of DMAX and OPE, OXA and OPE, or DMAO and OPE. (**b**) FT-IR spectra of the single compounds and mixtures of DMAX and OPE, OXA and OPE, DMAO and OPE. The key peak of Schiff base is marked with an arrow. (**c**) ^13^C-NMR spectra of single compounds and mixtures of DMAX and OA, OXA and OA, or DMAO and OA. (**d**) FT-IR spectra of single compounds and mixtures of DMAX and OA, OXA and OA, or DMAO and OA. (**e**) ^13^C-NMR spectra of DMAX plus OPE with NaCl (500 mM). The spectrum of the mixture at 0 mM NaCl was used as control. Dotted squares indicate the region of the ketone group in ^13^C-NMR spectra and the imine bond in FT-IR spectra. (**f**) Preparation of lipid-coated wells to study the binding of fluorescein and DMAX-FITC. (**g**) Relative fluorescence intensities of DMAX-FITC on different membranes normalized to uncoated wells. The fluorescein result was subtracted to obtain the difference. The data are mean ± standard error of mean (SEM) (*n* = 8); *p* value calculated by Student’s *t*-test.

The interaction of DMAX with the phosphatidylethanolamine headgroup (O-phosphoryl ethanolamine, OPE) was evaluated by ^13^C-NMR and FT-IR measurements. ^13^C-NMR was employed because of the overlap of the characteristic peak related to Schiff base with other peaks and the difficulty in its quantitative analysis in the ^1^H-NMR spectra (Figure S1). While ^13^C-NMR is typically not a quantitative technique under standard settings because of the Nuclear Overhauser Effect (NOE) and the long spin-lattice relaxation times (T1) for carbon nuclei [43,44], comparing the relative changes in the same peak before and after the reaction can provide semi-quantitative information when experimental conditions are identical. For this experiment, we used 4-oxopentanoic acid (OXA) and 5-(dimethylamino)-5-oxopentanoic acid (DMAO) as control ligands. OXA contains only a ketone group and lacks the dimethylamine moiety, whereas DMAO has both a carbonyl and a dimethylamine group, but these are linked through an amide bond. Moreover, we used n-octylamine (OA) as a control target lacking the phosphate group. In the ^13^C-NMR spectra analysis, the ketone carbon resonance of free DMAX at 225 ppm disappeared upon mixing with OPE, consistent with the formation of a Schiff base. Control ligands OXA and DMAO mixed with OPE did not exhibit this spectral change (Figure 3a). Also, none of the ligands, including DMAX, reacted with OA (Figure 3c). These results suggest that the tertiary amine in DMAX and the phosphate in OPE are essential for interaction.

In the FT-IR spectra analysis, we found that the mixture of DMAX and OPE presented a new peak at 1672 cm^−1^ (Figure 3b), which corresponds to C=N stretching, consistent with a Schiff base formation. This spectral change was not observed in the mixtures of OXA or DMAO with OPE, nor in any ligand mixed with OA (Figure 3d), confirming that both the tertiary amine in DMAX and the phosphate group in OPE are required for covalent engagement. The FT-IR data therefore corroborate the ^13^C-NMR results, supporting the ligand mechanism.

To investigate whether electrostatic interactions contribute to Schiff base formation between DMAX and OPE, we tested the effect of ionic strength on their interaction. NaCl (500 mM) was added to the DMAX-OPE mixture to disrupt potential electrostatic attraction. Under this condition the intensity of the ketone carbon peak of DMAX remained higher than that at 0 mM NaCl (Figure 3e), indicating that the Schiff base formation was affected at high ionic strength. These results indicate the importance of the electrostatic interaction between the protonated dimethylamine group of DMAX with the negatively charged phosphate of OPE for promoting the covalent bonding.

To determine whether DMAX selectively recognizes PE in biological membranes, DMAX was conjugated to fluorescein 5-isothiocyanate (FITC) via an ethylenediamine linker. Successful conjugation was confirmed by ^1^H-NMR (Figure S2). We then assessed the binding of the DMAX-FITC conjugate using lipid-coated well plates with varying compositions (Figure 3f). After 1 h incubation, the wells were washed, and the remaining fluorescence intensity was quantified and normalized to that of free FITC. The results showed that the intensity of DMAX-FITC increased with the PE content of the membranes (Figure 3g). Moreover, DMAX preferentially recognizes PE compared to other lipids, i.e., PC, amine containing PS and TA, and amine lacking DP. Particularly, DMAX-FITC showed no detectable binding to PC-only membranes. Moreover, its binding to amino-containing membranes lacking a phosphate group (TA) was 2-fold lower than that to PE-containing membranes. These results confirm that DMAX selectively engages its target PE in lipid membranes, supporting its potential for bacterial membrane targeting.

### 3.3. Binding Ability of DMAX for E. coli

**Figure 4:**
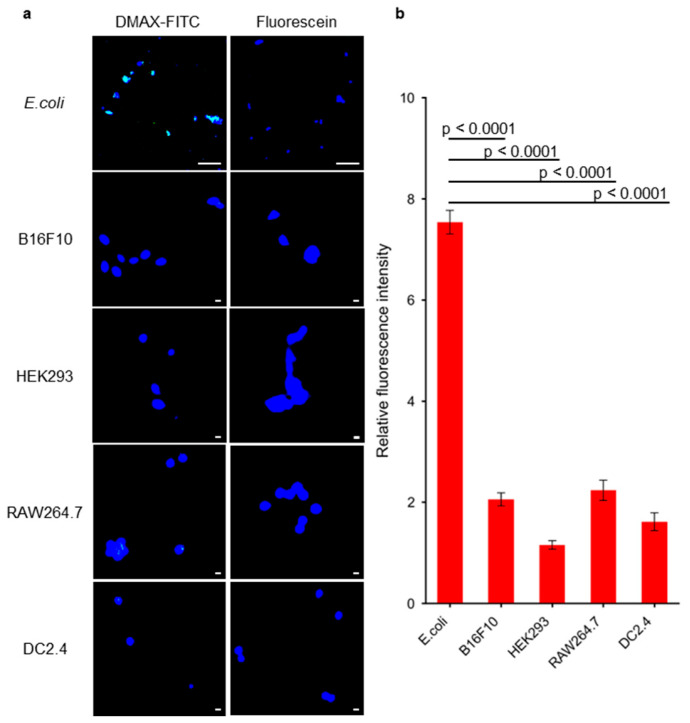
(**a**) Confocal microscopy images of DMAX-FITC or fluorescein (green) in *E. coli*, B16F10, HEK293, RAW264.7 and DC2.4 cells. The cell nuclei were stained with Hoechst (blue). Scale bar = 10 µm for *E. coli*, and 100 µm for B16F10, HEK293, RAW264.7 or DC2.4. (**b**) Quantification of colocalization of DMAX-FITC or fluorescein with bacteria or mammalian cells by measuring FITC intensity from 10 cells per group, normalized to controls of each group. The data are mean ± SEM, *p* value calculated by one-way ANOVA.

The interaction of DMAX-FITC and bacteria was evaluated by fluorescence confocal microscopy. Live *E. coli* cells were incubated for 1 h with free fluorescein or DMAX-FITC (Figure 4a). After washing, the DMAX-FITC treated cells showed stronger fluorescent signals compared to the cells treated with free fluorescein (Figure 4a). Quantitative analysis of Hoechst-stained cells and DMAX-FITC at 40 bacterial ROIs showed 7-fold greater fluorescence intensity for cells treated with DMAX-FITC compared with free fluorescein (*p* < 0.0001; Figure S3). Moreover, the off-target activity was evaluated in mammalian cells (Figure 4a). Murine melanoma cells (B16F10), human embryonic kidney epithelial cells (HEK293), murine macrophage-like cells (RAW264.7), and murine dendritic cells (DC2.4) were incubated with fluorescein or DMAX-FITC for 1 h. After washing, DMAX-FITC produced slightly higher cellular fluorescence than free fluorescein (Figure 4b), indicating limited non-specific uptake. Normalizing DMAX-FITC fluorescence intensity to fluorescein fluorescence intensity revealed that DMAX-FITC binding to bacteria was strongly enhanced, showing around a 4-fold increase in fluorescence intensity, underscoring its preferential bacterial targeting. These findings indicate that DMAX exhibits strong selectivity for bacteria, supporting its feasibility as a bacteria-targeted delivery system under physiological conditions.

### 3.4. Bacterial Growth Inhibition Assay

**Figure 5:**
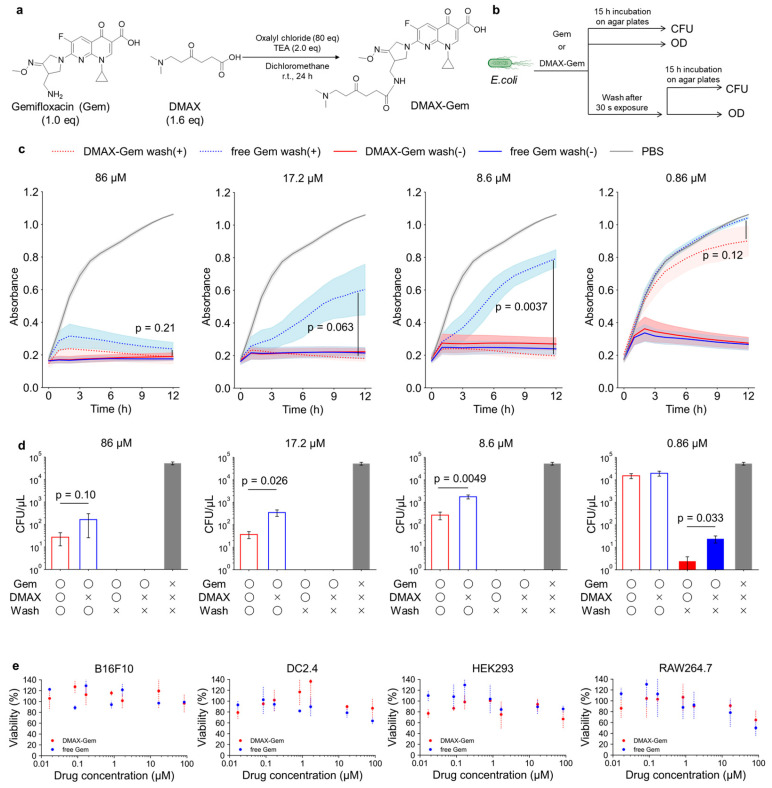
(**a**) Preparation of DMAX-Gem. (**b**) Experimental scheme of the bacterial growth inhibition assay. (**c**) *E. coli* treated with DMAX-Gem without wash (red), free Gem without wash (blue), DMAX-Gem with wash (dashed red), free Gem with wash (dashed blue), or PBS (gray) at 86, 17.2, 8.6, or 0.86 µM. The growth was measured as the absorbance over time. The data are mean ± SEM (*n* = 3); *p* values calculated with one-tailed Student’s *t*-test. (**d**) CFU assay of *E. coli* after the same treatments. The data are mean ± SEM (*n* = 5); *p* values calculated with one-tailed Student’s *t*-test. (**e**) CCK-8 assays of DMAX-Gem (red) or free Gem (blue) against mammalian cells (B16F10, DC2.4, HEK293, and RAW264.7). The data are mean ± SEM (*n* = 3).

We next applied DMAX to a clinically relevant drug scaffold to determine whether it can endow antibiotics with PE-targeting capability and thereby improve their antibacterial performance. We selected Gem, often classified as a fourth-generation fluoroquinolone, because it has a broad-spectrum potency against Gram-negative pathogens and an intracellular mechanism of action, i.e., DNA-gyrase-targeted bactericidal effects [45]. We reasoned that equipping Gem with PE-targeting would increase local drug concentration at bacterial surfaces, improving retention and efficacy. Since the primary amine of Gem is dispensable for antibacterial activity of fluoroquinolones [46], we used it as the site to attach DMAX (Figure 5a). Successful conjugation of DMAX to Gem was confirmed by ^1^H-NMR and TOF-MS (Figure S4).

We compared the efficacy of the positive control Gem with that of DMAX-Gem against *E. coli* under both continuous and short-pulse exposure to mimic drug clearance (Figure 5b). Under continuous exposure, both Gem and DMAX-Gem fully suppressed bacterial growth at the studied doses (Figure 5c). In both the absorbance and CFU measurements, the groups that were not washed after treatment exhibited complete growth inhibition at concentrations higher than 8.6 µM (CFU = 0; absorbance lower than 1.3-fold of initial values). However, CFU analysis at the lowest concentration (0.86 µM, Figure 5d) indicated that DMAX-Gem was more effective than Gem, achieving 10-fold lower CFU/µL. Remarkably, the short-pulse exposure revealed the true advantage of DMAX. A 3 min incubation followed by washing was enough for DMAX-Gem to inhibit bacterial growth at 8.6 µM, while free Gem failed even at 17.2 µM. Consistent with this, evaluation of viability by CFU confirmed that DMAX-Gem significantly reduced colony formation after washing (Figure 5d). At 86 µM, 17.2 µM, and 8.6 µM, the CFU values for DMAX-Gem were approximately one order of magnitude lower than those for free Gem, demonstrating enhanced antibacterial activity under rapid clearance conditions.

Importantly, DMAX conjugation did not compromise mammalian cell compatibility. Cell viability after the treatment with DMAX-Gem remained comparable to that of free Gem (positive control) up to 86 µM across multiple mammalian cell lines, including B16F10 melanoma cells, DC2.4 dendritic cells, HEK293 cells, and RAW264.7 macrophages (Figure 5e). Notably, DMAX-Gem-treated groups retained approximately 90% cell viability at 17.2 µM, a concentration at which bactericidal efficacy was significantly enhanced relative to free Gem. Previously reported PE-targeting systems, including duramycin and cinnamycin [19], urea-functionalized crown ethers [21,22,23], and cyclotides [24,25], exhibit cytotoxicity to mammalian cells due to their membrane-disruptive mechanisms. In contrast, DMAX-modified antibiotics did not increase mammalian cell toxicity even at bactericidal concentrations, highlighting the non-lytic and selective nature of the DMAX-mediated targeting strategy.

This selectivity also distinguishes DMAX from other covalent PE-targeting approaches, such as iminoboronate-based chemistries [47]. While iminoboronate formation relies on reversible covalent interactions with primary amines, these amines are broadly distributed across proteins, lipids, and cell-surface biomolecules, leading to promiscuous binding and limited membrane specificity. By contrast, DMAX targets the PE headgroup through a combination of reversible covalent interaction and membrane-context–dependent stabilization, enabling selective bacterial membrane anchoring without off-target engagement of mammalian cells.

As a small synthetic molecule, DMAX offers several practical advantages over medium- or macromolecule-based targeting systems, such as antibodies and peptides. DMAX exhibits high chemical stability, allowing long-term storage (Figure S5) and compatibility with diverse chemical conjugation strategies without loss of activity. It also remains stable under biological conditions, in contrast to antibodies and peptides that are often susceptible to environmental or enzymatic degradation [7,8]. Moreover, the molecular weight of DMAX (174 Da) is orders of magnitude smaller than that of antibodies and substantially smaller than peptides, reducing the likelihood of adversely altering diffusion, tissue penetration and pharmacokinetic profiles of the parent drug through the drastic change in the molecular weight [48,49].

## 4. Conclusions

In summary, we presented a self-stabilizing ligand strategy to deliver antibiotics selectively to bacterial membranes via minimal chemical modification. By integrating reversible covalent bonds with electrostatic interactions, DMAX exploits intrinsic lipid chemistry for superior bacterial affinity. This self-stabilizing engagement yields exceptional selectivity, enduring membrane retention and providing potent antibacterial efficacy, even after a 3 min exposure and rigorous washing. Ultimately, DMAX converts transient interactions into selective anchors, creating a modular platform that can be extended from antibiotics to targeted diagnostics, probes, nanomedicines and immune effectors.

## 5. Patents

The University of Tokyo; Cabral, H.; Masuda, K.; Takai, M.; Masuda, T.; Nakagawa, Y. Bacterial Recognition Ligand. Japanese Patent Application JP 2024-084259, filed 23 May 2024 [50].

## Data Availability

The data presented in this study are available upon request from the corresponding author.

## Supplementary Materials

The following supporting information can be downloaded at https://www.mdpi.com/article/10.3390/pharmaceutics18010071/s1: Computational method presents the code used for the computational analyses; Figure S1: Study of the Schiff base formation by ^1^H-NMR; Figure S2: The reaction scheme and the ^1^H-NMR spectra of the FITC conjugates; Figure S3. DMAX selectively binds to bacteria; Figure S4. The ^1^H-NMR and TOF-MS spectra of the Gemifloxacin conjugate. Figure S5. The ^1^H-NMR and TOF-mass spectra of the DMAX.

## Abbreviations

The following abbreviations are used in this manuscript:

AEPP	[(2R)-3-[2-aminoethoxy(hydroxy)phosphoryl]oxy-2-propanoyloxypropyl] propanoate
AEPPH	Protonated AEPP
ANOVA	analysis of variance
CFU	Colony forming units
CLSM	confocal laser scanning microscopy
DMAX	6-dimethylamino-4-ketohexanoic acid
DMAO	5-(dimethylamino)-5-oxopentanoic acid
DMEM	Dulbecco’s Modified Eagle Medium
DMF	dimethylformamide
DMSO	dimethyl sulfoxide
DMT-MM	4-(4,6-dimethoxy-1,3,5-triazin-2-yl)-4-methylmorpholinium chloride
DPC	1,2-dibutyrylphosphatidylcholine
EDA	ethylenediamine
EDMAX	Ethyl-DMAX
EDMAXH	Protonated EDMAX
ESI-TOF MS	electrospray ionization–time-of-flight mass spectrometry
FBS	fetal bovine serum
FITC	fluorescein 5-isothiocyanate
FT-IR	Fourier-transform infrared spectroscopy
Gem	gemifloxacin
LB	Luria–Bertani medium
NMR	nuclear magnetic resonance
NOE	nuclear Overhauser effect
OA	n-octylamine
OD	optical density
OPE	O-phosphoryl ethanolamine
OXA	4-oxopentanoic acid
PBS	phosphate-buffered saline
PC	phosphatidylcholine
PE	phosphatidylethanolamine
PFA	paraformaldehyde
PS	phosphatidylserine
RFU	relative fluorescence units
ROI	region of interest
RPMI	Roswell Park Memorial Institute medium
SEM	standard error of the mean
SM	sphingomyelin
TA	tetradecylamine
TEA	triethylamine
