# Supplementary material for: Self-Stabilizing Covalent Ligand Targets Bacterial Phosphatidylethanolamine and Enhances Antibiotic Efficacy
Source: Pharmaceutics. 2026 Jan 5;18(1):71. doi: 10.3390/pharmaceutics18010071 (PMC12845377; doi:10.3390/pharmaceutics18010071)
Supplement: Supplementary file 1 [file pharmaceutics-18-00071-s001.zip › pharmaceutics-4050109-supplementary.pdf]

Supporting Information

Self-stabilizing covalent ligand targets bacterial phosphatidylethanolamine and enhances antibiotic efficacy

Keita Masuda[1], Yasuhiro Nakagawa[1], Quentin Boussau[2], Emilie Chabert[2], Tsukuru Masuda[1], Jerome Bonnet[2], Tatsuya Inukai[3], Shigeki Nakamura[3], Madoka Takai[1,4], Diego Cattoni[2] and Horacio Cabral[1*]

1. Department of Bioengineering, Graduate School of Engineering, The University of Tokyo, 7-3-1 Hongo, Bunkyo-ku, Tokyo 113-8656, Japan
2. Centre de Biologie Structurale, CNRS UMR 5048, INSERM U1054, Université de Montpellier, 29 rue de Navacelles, 34090, Montpellier, France
3. Department of Microbiology, Tokyo Medical University, 6-1-1 Shinjuku, Shinjuku-ku, Tokyo 160-8402, Japan.
4. Research Center for Water Environment Technology, School of Engineering, The University of Tokyo, 7-3-1 Hongo, Bunkyo-ku, Tokyo 113-8656, Japan

*Correspondence to:

E-mail: horacio@bmw.t.u-tokyo.ac.jp

Contents

1. ***Computational method*** 3

2. Results 10

2.1 Interaction between DMAX and OPE 10

2.2 Preparation of FITC-conjugate 12

2.3 Binding ability of DMAX for E. coli 13

2.4 Preparation of Gemifloxacin conjugate 14

REFERENCES 15

1. *Computational method*

The code below was used for NWChem software in Linux terminal to conduct the calculation above. In the linux terminal, a project file name as "ffunction" was created in the working directory and the code below was written there to run the NWChem software. The produced fminus.cube and fplus.cube files were used to visualize the reactivity of each atom in the molecule by VESTA software. The fminus.cube was used to visualize the nucleophilicity and the fplus electrophilicity. The produced tsv file contains the quantitative information of fminus and fplus profile.

```
# ===== strict mode (portable) =====
set -eu
if ( set -o 2>/dev/null | grep -q '^pipefail' ) 2>/dev/null; then set -o pipefail; fi

# ===== user controls =====
GRID_POINTS=81
MARGIN_A=3.0
SEED_XC="pbe96"
REF_XC="b3lyp"
BASIS_DESC="6-31g*"
SCF_ITER_SEED=220
SCF_ITER_REF=250
MEM_SEED_MB=1200
MEM_REF_MB=2200
MEM_DPLOT_MB=800

say(){ printf "\n\033[1;36m[%s]\033[0m %s\n" "$1" "$2"; }
die(){ echo -e "\n[ERR] $*" >&2; exit 1; }
need(){ command -v "$1" >/dev/null 2>&1 || die "Missing: $1"; }

need obabel; need nwchem; need python3; need awk; need grep

basis_block(){ cat <<EOF
basis
  * library ${BASIS_DESC}
end
EOF
}

# ===== DPLOT input writer =====
make_dplot_nw(){
  local tag="$1" vec="$2" outcube="$3" xmin="$4" xmax="$5" nxsp="$6" ymin="$7" ymax="$8" nysp="$9" zmin="${10}" zmax="${11}" nzsp="${12}"
  cat > "dplot_${tag}.nw" <<EOF
start dplot_${tag}
memory total ${MEM_DPLOT_MB} mb
title "rho_${tag}"
```

```
geometry units angstrom nocenter noautoz noautosym
$(awk 'NR>2 && NF>=4 {printf("  %-2s % .8f % .8f % .8f\n",$1,$2,$3,$4)}' "$XYZ")
end
$(basis_block)
dplot
  TITLE rho_${tag}
  VECTORS ${vec}
  LIMITXYZ
    ${xmin} ${xmax} ${nxsp}
    ${ymin} ${ymax} ${nysp}
    ${zmin} ${zmax} ${nzsp}
  SPIN total
  GAUSSIAN
  OUTPUT ${outcube}
end
task dplot
EOF
}

# ===== main =====
# default to Sample.mol if no args
if [[ $# -lt 1 ]]; then
  set -- "Sample.mol"
fi

for MOL in "$@"; do
  [[ -s "$MOL" ]] || die "No such file: $MOL"
  STEM=$(basename "$MOL" .mol)
  say "MOL" "$STEM"

  # 1) mol -> xyz
  XYZ="${STEM}.xyz"
  say "OBABEL" "to XYZ"
  obabel "$MOL" -O "$XYZ" --gen3d -h
  [[ -s "$XYZ" ]] || die "obabel failed to write $XYZ"

  # 2) geom.nw
  say "GEOM" "geom.nw"
  {
    echo "geometry units angstrom nocenter noautoz noautosym"
    awk 'NR>2 && NF>=4 {printf("  %-2s % .8f % .8f % .8f\n",$1,$2,$3,$4)}' "$XYZ"
    echo "end"
  } > geom.nw

  # 3) spin diagnostics（neutral/anion/cation）
  say "DIAG" "electrons & spin"
  eval "$(python3 - "$XYZ" <<'PY'
import sys,re,pathlib
```

```
xyzp=pathlib.Path(sys.argv[1])
t=xyzp.read_text(errors='ignore').splitlines()
atoms=[re.split(r'\s+',L.strip())[0].capitalize() for L in t[2:] if L.strip()]
Z={"H":1,"He":2,"Li":3,"Be":4,"B":5,"C":6,"N":7,"O":8,"F":9,"Ne":10,"Na":11,"Mg":12,
"Al":13,"Si":14,"P":15,"S":16,"Cl":17,"Ar":18,"K":19,"Ca":20,"Sc":21,"Ti":22,"V":23,"Cr":24,"Mn":25
,
"Fe":26,"Co":27,"Ni":28,"Cu":29,"Zn":30,"Ga":31,"Ge":32,"As":33,"Se":34,"Br":35,"Kr":36,"Rb":37,"
Sr":38,
"Y":39,"Zr":40,"Nb":41,"Mo":42,"Tc":43,"Ru":44,"Rh":45,"Pd":46,"Ag":47,"Cd":48,"In":49,"Sn":50,"
Sb":51,
"Te":52,"I":53,"Xe":54,"Cs":55,"Ba":56}
Ztot=sum(Z[a] for a in atoms)
def rec(q):
   e=Ztot-q
   mult=2 if e%2 else 1
   odft=1 if e%2 else 0
   return mult,odft
mN,oN=rec(0); mA,oA=rec(-1); mC,oC=rec(+1)
print(f"MULT_N={mN}");        print(f"ODFT_N={oN}");        print(f"MULT_A={mA}");
print(f"ODFT_A={oA}"); print(f"MULT_C={mC}"); print(f"ODFT_C={oC}")
PY
)"
  echo "      MULT_N=$MULT_N   ODFT_N=$ODFT_N   |   MULT_A=$MULT_A
ODFT_A=$ODFT_A | MULT_C=$MULT_C ODFT_C=$ODFT_C"

  # 4) Neutral seed
  if [[ ! -s sp_N_seed.movecs ]]; then
   say "RUN" "Neutral seed (${SEED_XC})"
   cat > sp_N_seed.nw <<EOF
start sp_N_seed
memory total ${MEM_SEED_MB} mb
title "Neutral seed"
$(cat geom.nw)
$(basis_block)
charge 0
dft
  xc ${SEED_XC}
  grid medium
  mult ${MULT_N}
  $( (( ODFT_N==1 )) && echo "odft" )
  iterations ${SCF_ITER_SEED}
  convergence energy 1e-6
  vectors atomic
  vectors output sp_N_seed.movecs
end
task dft energy
EOF
   nwchem sp_N_seed.nw > sp_N_seed.out 2>&1
```

```
    grep -q "Total DFT energy" sp_N_seed.out || die "Neutral seed failed"
  fi

  # 5) Neutral refine
  if [[ ! -s sp_N_ref.movecs ]]; then
    say "RUN" "Neutral refine (${REF_XC})"
    cat > sp_N_ref.nw <<EOF
start sp_N_ref
memory total ${MEM_REF_MB} mb
title "Neutral refine"
$(cat geom.nw)
$(basis_block)
charge 0
dft
  xc ${REF_XC}
  mulliken
  grid xfine
  mult ${MULT_N}
  $( (( ODFT_N==1 )) && echo "odft" )
  iterations ${SCF_ITER_REF}
  convergence energy 1e-6
  vectors input sp_N_seed.movecs
  vectors output sp_N_ref.movecs
end
task dft energy
EOF
    nwchem sp_N_ref.nw > sp_N_ref.out 2>&1
    grep -q "Total DFT energy" sp_N_ref.out || die "Neutral refine failed"
  fi

  # 6) Anion refine
  if [[ ! -s sp_anion_ref.movecs ]]; then
    say "RUN" "Anion refine (${REF_XC})"
    cat > sp_anion_ref.nw <<EOF
start sp_anion_ref
memory total ${MEM_REF_MB} mb
title "Anion refine"
$(cat geom.nw)
$(basis_block)
charge -1
dft
  xc ${REF_XC}
  mulliken
  grid xfine
  mult ${MULT_A}
  $( (( ODFT_A==1 )) && echo "odft" )
  iterations 160
  convergence energy 1e-6
```

```
  vectors output sp_anion_ref.movecs
end
task dft energy
EOF
    nwchem sp_anion_ref.nw > sp_anion_ref.out 2>&1
    grep -q "Total DFT energy" sp_anion_ref.out || die "Anion refine failed"
  fi

  # 7) Cation refine
  if [[ ! -s sp_cation_ref.movecs ]]; then
    say "RUN" "Cation refine (${REF_XC})"
    cat > sp_cation_ref.nw <<EOF
start sp_cation_ref
memory total ${MEM_REF_MB} mb
title "Cation refine"
$(cat geom.nw)
$(basis_block)
charge +1
dft
  xc ${REF_XC}
  mulliken
  grid xfine
  mult ${MULT_C}
  $( (( ODFT_C==1 )) && echo "odft" )
  iterations 160
  convergence energy 1e-6
  vectors output sp_cation_ref.movecs
end
task dft energy
EOF
    nwchem sp_cation_ref.nw > sp_cation_ref.out 2>&1
    grep -q "Total DFT energy" sp_cation_ref.out || die "Cation refine failed"
  fi

  # 8) LIMITXYZ from xyz（81 points→spacings=80、±3 Å マージン）
  read XMIN XMAX NXSP YMIN YMAX NYSP ZMIN ZMAX NZSP < <(python3 - "$GRID_POINTS" "$MARGIN_A" "$XYZ" <<'PY'
import sys, pathlib
pts=int(sys.argv[1]); pad=float(sys.argv[2]); xyzp=pathlib.Path(sys.argv[3])
t=xyzp.read_text(errors='ignore').splitlines()[2:]
xs=[];ys=[];zs=[]
for L in t:
    if not L.strip(): continue
    _,x,y,z=L.split()[:4]
    xs.append(float(x)); ys.append(float(y)); zs.append(float(z))
xmin=min(xs)-pad; xmax=max(xs)+pad
ymin=min(ys)-pad; ymax=max(ys)+pad
zmin=min(zs)-pad; zmax=max(zs)+pad
```

```
sp=pts-1
print(f"{xmin} {xmax} {sp} {ymin} {ymax} {sp} {zmin} {zmax} {sp}")
PY
)
  say "DPLOT" "X[${XMIN},${XMAX}] Y[${YMIN},${YMAX}] Z[${ZMIN},${ZMAX}] spacings=${NXSP}"

  # 9) DPLOT (manual-compliant)
  make_dplot_nw "N" "sp_N_ref.movecs" "rho_N.cube" "$XMIN" "$XMAX" "$NXSP" "$YMIN" "$YMAX" "$NYSP" "$ZMIN" "$ZMAX" "$NZSP"
  make_dplot_nw "anion" "sp_anion_ref.movecs" "rho_anion.cube" "$XMIN" "$XMAX" "$NXSP" "$YMIN" "$YMAX" "$NYSP" "$ZMIN" "$ZMAX" "$NZSP"
  make_dplot_nw "cation" "sp_cation_ref.movecs" "rho_cation.cube" "$XMIN" "$XMAX" "$NXSP" "$YMIN" "$YMAX" "$NYSP" "$ZMIN" "$ZMAX" "$NZSP"

  nwchem dplot_N.nw      > dplot_N.out      2>&1 || die "DPLOT neutral failed"
  nwchem dplot_anion.nw  > dplot_anion.out  2>&1 || die "DPLOT anion failed"
  nwchem dplot_cation.nw > dplot_cation.out 2>&1 || die "DPLOT cation failed"

  # 10) Fukui cubes（ヘッダ維持）
  say "FUKUI" "write fplus/fminus cubes"
  python3 - <<'PY'
import numpy as np
def rc(p):
    with open(p,'r') as f:
        h=[f.readline(),f.readline(),f.readline()]
        ax=[f.readline(),f.readline(),f.readline()]; h+=ax
        nat=int(h[2].split()[0])
        atoms=[f.readline() for _ in range(abs(nat))]; h+=atoms
        dat=np.fromfile(f,sep=' ')
    nx=int(ax[0].split()[0]); ny=int(ax[1].split()[0]); nz=int(ax[2].split()[0])
    assert dat.size==nx*ny*nz
    return h,dat
def wc(p,h,d):
    with open(p,'w') as g:
        for L in h: g.write(L if L.endswith('\n') else L+'\n')
        for i,v in enumerate(d,1):
            g.write(f"{v: .6E} ")
            if i%6==0: g.write("\n")
        if len(d)%6: g.write("\n")
hN,rN=rc("rho_N.cube"); _,rA=rc("rho_anion.cube"); _,rC=rc("rho_cation.cube")
wc("fplus.cube",  hN, rA-rN)
wc("fminus.cube", hN, rN-rC)
print("[OK] fplus.cube & fminus.cube")
PY

  # 11) Condensed Fukui（Voronoi 分割）
  say "CONDENSE" "Voronoi→TSV"
```

```
 python3 - <<'PY'
import numpy as np, pathlib
def read_cube(p):
  with open(p,'r') as f:
    f.readline(); f.readline()
    l3=f.readline().split(); nat=int(l3[0]); origin=np.array(list(map(float,l3[1:4])))
    ax1=list(map(float,f.readline().split())); ax2=list(map(float,f.readline().split()));
ax3=list(map(float,f.readline().split()))
    nx,vx=int(ax1[0]),np.array(ax1[1:4]); ny,vy=int(ax2[0]),np.array(ax2[1:4]);
nz,vz=int(ax3[0]),np.array(ax3[1:4])
    atoms=[]
    for _ in range(abs(nat)):
      toks=f.readline().split(); Z=int(float(toks[0]))
      if len(toks)>=5: x,y,z=map(float,toks[-3:])
      else: x,y,z=map(float,toks[1:4])
      atoms.append((Z,np.array([x,y,z])))
    data=np.fromfile(f,sep=' ')
  nx,ny,nz=abs(nx),abs(ny),abs(nz)
  assert data.size==nx*ny*nz
  return dict(origin=origin,vx=vx,vy=vy,vz=vz,nx=nx,ny=ny,nz=nz,atoms=atoms,data=data)
def vvol(vx,vy,vz): import numpy as np; return
abs(np.linalg.det(np.column_stack([vx,vy,vz])))
Z2SYM={1:"H",6:"C",7:"N",8:"O",9:"F",15:"P",16:"S",17:"Cl",35:"Br",53:"I"}
def sym(Z): return Z2SYM.get(Z,f"Z{Z}")
N=read_cube("rho_N.cube"); A=read_cube("rho_anion.cube");
C=read_cube("rho_cation.cube")
for k in("nx","ny","nz"): assert N[k]==A[k]==C[k]
for k in("origin","vx","vy","vz"):
  import numpy as np
  assert np.allclose(N[k],A[k]) and np.allclose(N[k],C[k])
nx,ny,nz=N["nx"],N["ny"],N["nz"]
rhoN=N["data"].reshape(nx,ny,nz); rhoA=A["data"].reshape(nx,ny,nz);
rhoC=C["data"].reshape(nx,ny,nz)
fplus=rhoA-rhoN; fminus=rhoN-rhoC
dV=vvol(N["vx"],N["vy"],N["vz"])
centers=np.stack([p for _,p in N["atoms"]]); Nat=len(N["atoms"])
accp=np.zeros(Nat); accm=np.zeros(Nat)
def slab_coords(o,vx,vy,vz,nx,ny,k0,k1):
  o4=o.reshape(1,1,1,3); vx4=vx.reshape(1,1,1,3); vy4=vy.reshape(1,1,1,3);
vz4=vz.reshape(1,1,1,3)
  ii=np.arange(nx,dtype=float).reshape(nx,1,1,1);
jj=np.arange(ny,dtype=float).reshape(1,ny,1,1);
kk=np.arange(k0,k1,dtype=float).reshape(1,1,k1-k0,1)
  return o4+ii*vx4+jj*vy4+kk*vz4
SLAB=max(4,min(nz,16))
for k0 in range(0,nz,SLAB):
  k1=min(k0+SLAB,nz); R=slab_coords(N["origin"],N["vx"],N["vy"],N["vz"],nx,ny,k0,k1)
  M=(k1-k0)*nx*ny; R2=R.reshape(M,3)
```

```
    d2=np.sum((R2[:,None,:]-centers[None,:,:])**2,axis=2); owner=np.argmin(d2,axis=1)
    fp=fplus[:,:,k0:k1].reshape(M); fm=fminus[:,:,k0:k1].reshape(M)
    np.add.at(accp,owner,fp*dV); np.add.at(accm,owner,fm*dV)
totp=float(accp.sum()); totm=float(accm.sum())
with open("fukui_from_cube_voronoi.tsv","w") as g:
    g.write("# Condensed Fukui from cubes (Voronoi)\n# idx\tel\tx\ty\tz\tf_plus\tf_minus\tdelta_f\n")
    for i,((Z,xyz),fp,fm) in enumerate(zip(N["atoms"],accp,accm),start=1):
        g.write(f"{i}\t{sym(Z)}\t{xyz[0]:.6f}\t{xyz[1]:.6f}\t{xyz[2]:.6f}\t{fp:.6f}\t{fm:.6f}\t{(fp-fm):.6f}\n")
print(f"[SUM] ∑f+={totp:.6f} ∑f-={totm:.6f}")
PY

  say "DONE" "$STEM → rho_*.cube, fplus/fminus.cube, fukui_from_cube_voronoi.tsv"
done

# ===== How to run the code =====
#run the code by "bash ffunction.sh" command.
```

2. Results

2.1 Interaction between DMAX and OPE

The interaction of DMAX or other control ligands and OPE was confirmed by ^{1}H-NMR as well.

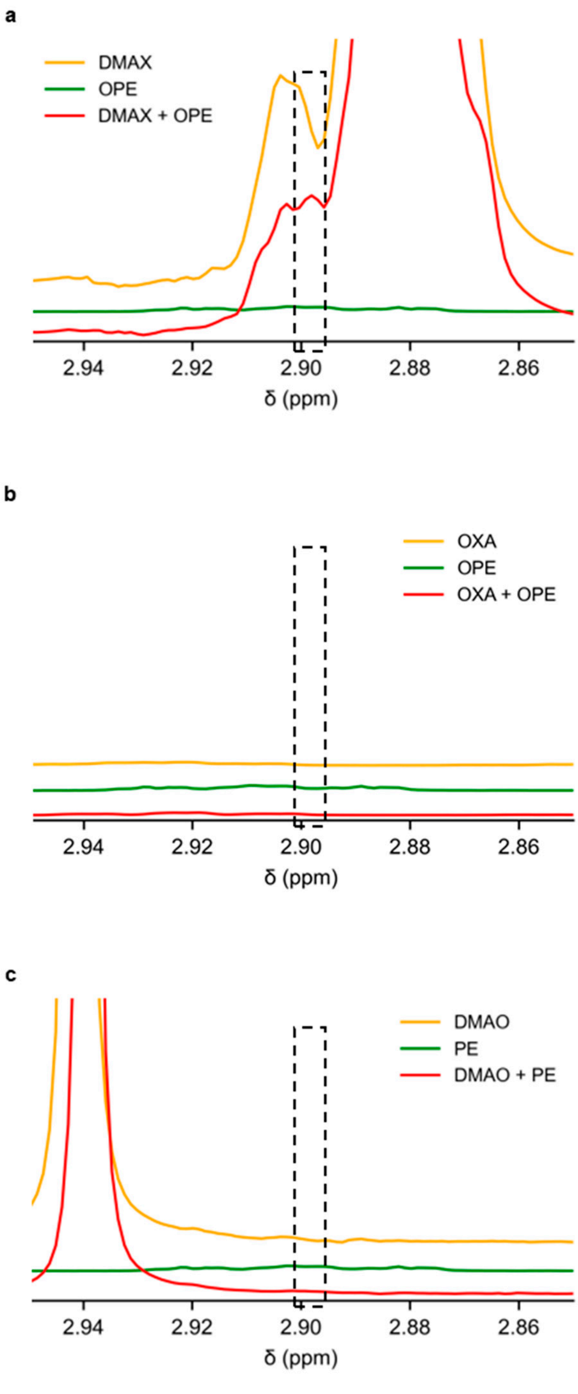

Figure S1. Study of the Schiff base formation by ^{1}H-NMR. The ^{1}H-NMR spectra of (a) DMAX and OPE, (b) OXA and OPE, and (c) DMAO and OPE. The orange line shows the free ligand, DMAX, OXA, or DMAO, the green line shows the OPE, and the red line shows the mixture of the ligand and the OPE. The dotted squares indicate the position of the Schiff base peak.

2.2 Preparation of FITC-conjugate

The FITC labeled DMAX was prepared by firstly modifying the FITC with diamine linker, and followed amide coupling reaction.

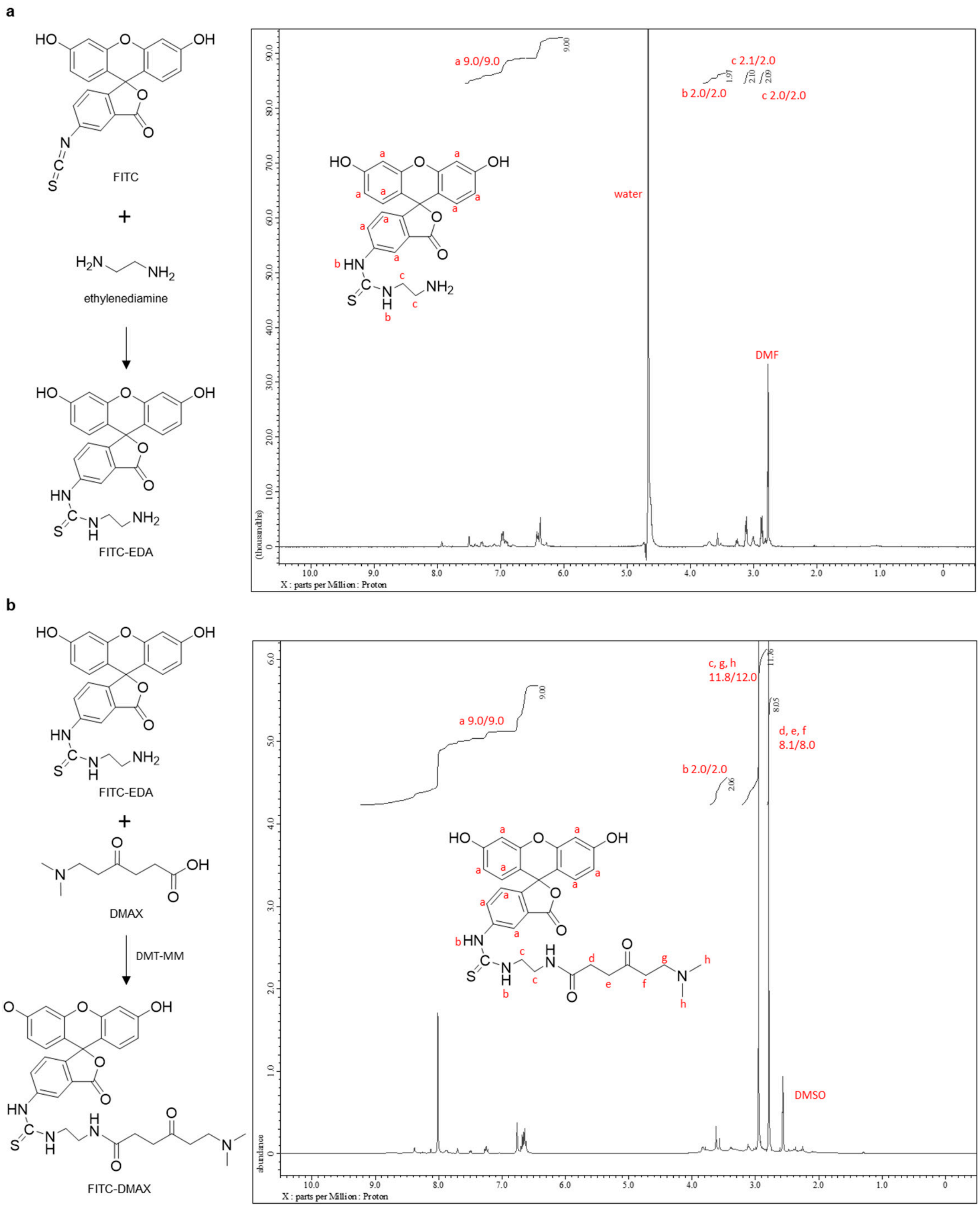

Figure S2. The reaction scheme and the ^{1}H-NMR spectra of the FITC conjugates. (a) The reaction scheme to functionalize the FITC with amine by ethylenediamine linker and the ^{1}H-NMR spectrum of the FITC-EDA. (b) The reaction scheme to conjugate the DMAX with FITC-EDA and the ^{1}H-NMR spectrum of the FITC-DMAX.

2.3 Binding ability of DMAX for E. coli

The binding ability of DMAX to *E. coli* was determined from the fluorescence intensity of 40 ROIs in comparison with the free fluorescein. The binding ability of DMAX to mammalian cells including B16F10, HEK293, RAW264.7 and DC2.4 was determined from the fluorescence intensity of 10 ROIs in comparison with the free fluorescein.

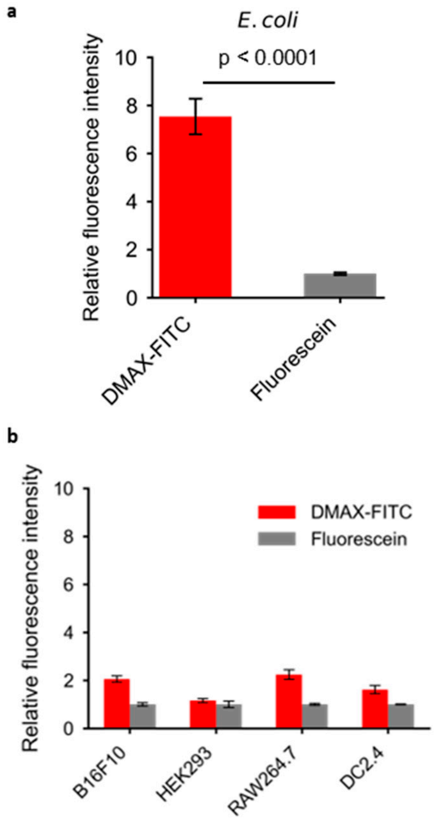

Figure S3. DMAX selectively binds to bacteria. Quantification of colocalization of DMAX-FITC or fluorescein with (a) *E. coli* and (b) mammalian cells including B16F10, HEK293, RAW264.7, and DC2.4 by measuring FITC intensity. For quantification, 40 ROIs per group were used for *E. coli* and 10 for mammalian cells. Values were normalized to fluorescein control. Data are mean ± SEM, analyzed by one-tailed t-test.

2.4 Preparation of Gemifloxacin conjugate

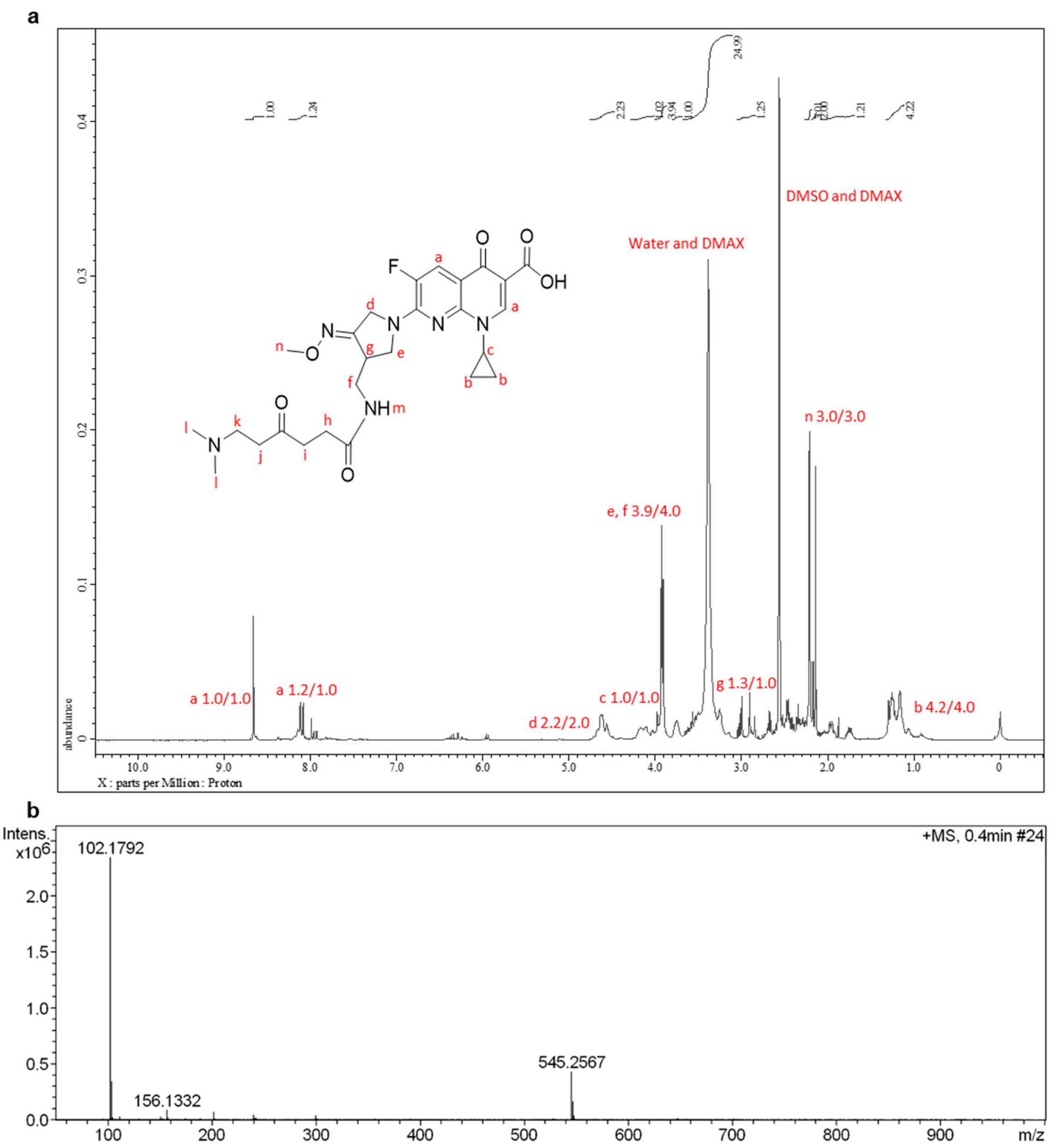

Figure S4. The ^{1}H-NMR and TOF-mass spectra of the gemifloxacin conjugate. (a) The ^{1}H-NMR spectrum of the DMAX-Gem. (b) The mass spectra of the DMAX-Gem.

2.5 Stability of DMAX

The long-term stability of DMAX was confirmed by ^{1}H-NMR and TOF-MS. The DMAX was kept for 5 months at room temperature, and ^{1}H-NMR and TOF-MS measurements were conducted. The result demonstrates the stability of DMAX during storage without any undesired peaks.

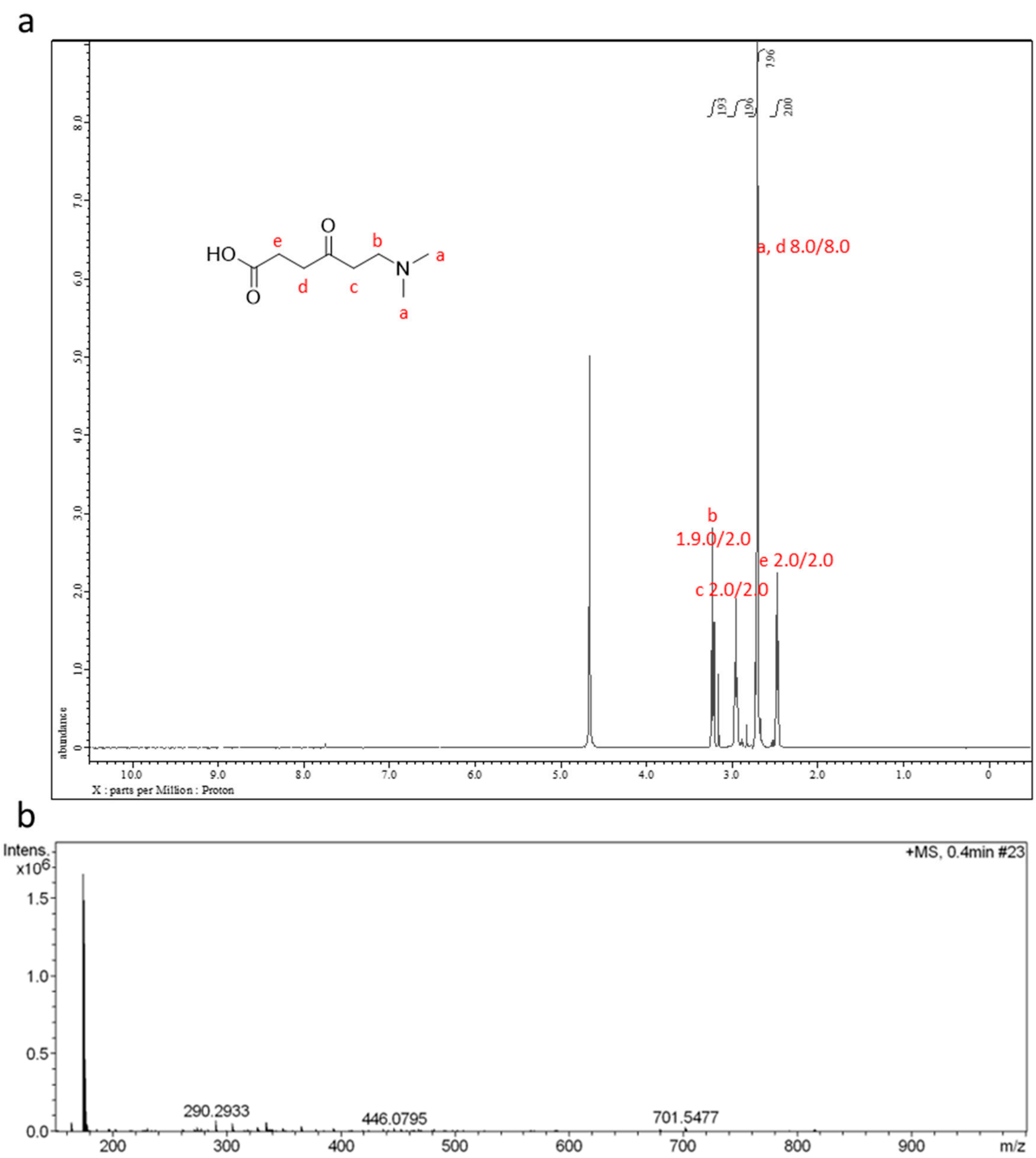

Figure S5. The ^{1}H-NMR and TOF-mass spectra of the DMAX. (a) The ^{1}H-NMR spectrum of the DMAX. (b) The mass spectra of the DMAX.

REFERENCES

(1) Seco, E. M.; Fernández, L. Á. Efficient Markerless Integration of Genes in the Chromosome of Probiotic *E. Coli* Nissle 1917 by Bacterial Conjugation. *Microb. Biotechnol.* **2022**, *15* (5), 1374–1391. https://doi.org/10.1111/1751-7915.13967.

(2) Valiev, M.; Bylaska, E. J.; Govind, N.; Kowalski, K.; Straatsma, T. P.; Van Dam, H. J. J.; Wang, D.; Nieplocha, J.; Apra, E.; Windus, T. L.; De Jong, W. A. NWChem: A Comprehensive and Scalable Open-Source Solution for Large Scale Molecular Simulations. *Comput. Phys. Commun.* **2010**, *181* (9), 1477–1489. https://doi.org/10.1016/j.cpc.2010.04.018.

(3) Calais, J. Density-functional Theory of Atoms and Molecules. R.G. Parr and W. Yang, Oxford University Press, New York, Oxford, 1989. IX + 333 Pp. Price £45.00. *Int. J. Quantum Chem.* **1993**, *47* (1), 101–101. https://doi.org/10.1002/qua.560470107.

(4) Yang, Weitao.; Mortier, W. J. The Use of Global and Local Molecular Parameters for the Analysis of the Gas-Phase Basicity of Amines. *J. Am. Chem. Soc.* **1986**, *108* (19), 5708–5711. https://doi.org/10.1021/ja00279a008.

(5) Contreras, R. R.; Fuentealba, P.; Galván, M.; Pérez, P. A Direct Evaluation of Regional Fukui Functions in Molecules. *Chem. Phys. Lett.* **1999**, *304* (5–6), 405–413. https://doi.org/10.1016/S0009-2614(99)00325-5.

(6) Kunishima, M.; Kawachi, C.; Hioki, K.; Terao, K.; Tani, S. Formation of Carboxamides by Direct Condensation of Carboxylic Acids and Amines in Alcohols Using a New Alcohol- and Water-Soluble Condensing Agent: DMT-MM. *Tetrahedron* **2001**, *57* (8), 1551–1558. https://doi.org/10.1016/S0040-4020(00)01137-6.

(7) Liu, M.; Xu, W.; Xu, L.; Zhong, G.; Chen, S.; Lu, W. Synthesis and Biological Evaluation of Diethylenetriamine Pentaacetic acid−Polyethylene Glycol−Folate: A New Folate-Derived, ^{99m}Tc-Based Radiopharmaceutical. *Bioconjug. Chem.* **2005**, *16* (5), 1126–1132. https://doi.org/10.1021/bc050122m.

(8) Yang, W.; Chen, P.; Boonstra, E.; Hong, T.; Cabral, H. Polymeric Micelles with pH-Responsive Cross-Linked Core Enhance In Vivo mRNA Delivery. *Pharmaceutics* **2022**, *14* (6), 1205. https://doi.org/10.3390/pharmaceutics14061205.
